# A Systematic Review Exploring the Effect of Human Immunodeficiency Virus on Cardiac Diseases

**DOI:** 10.7759/cureus.28960

**Published:** 2022-09-08

**Authors:** Mohammed J Alzubaidee, Raga Sruthi Dwarampudi, Sheena Mathew, Sumahitha Bichenapally, Vahe Khachatryan, Asmaa Muazzam, Chandani Hamal, Lakshmi Sai Deepak Reddy Velugoti, Godfrey Tabowei, Greeshma N Gaddipati, Maria Mukhtar, Safeera Khan

**Affiliations:** 1 Internal Medicine, California Institute of Behavioral Neurosciences & Psychology, Fairfield, USA; 2 Research, California Institute of Behavioral Neurosciences & Psychology, Fairfield, USA; 3 Pediatrics, California Institute of Behavioral Neurosciences & Psychology, Fairfield, USA; 4 Pathology Research, California Institute of Behavioral Neurosciences & Psychology, Fairfield, USA; 5 Internal Medicine/Family Medicine, California Institute of Behavioral Neurosciences & Psychology, Fairfield, USA

**Keywords:** hiv protease inhibitors, hiv-associated lipodystrophy syndrome, heart disease, heart failure, hiv

## Abstract

Human immunodeficiency virus (HIV) primarily affects the immune systems, which, if progressed, will lead to acquired immunodeficiency syndrome (AIDS). Currently, there is no effective cure for the disease, and patients are affected lifelong, but there are antiretroviral medications that can control the disease's symptoms and progression. In addition, taking precautions during sexual contact, especially in the male homosexual population, while handling the patient's bodily fluids such as blood and saliva, and during childbirth by an infected mother is necessary to prevent the transmission of the virus. We used 15 studies, including systematic reviews and meta-analyses, observation studies, randomized clinical trials, and comprehensive reviews, to determine how HIV interferes with heart disease, increasing morbidity and mortality. We have used specific inclusion and exclusion criteria, focusing on specified age groups within a particular timeline. Some of the included studies found that many side effects from antiretroviral drugs can impact heart conditions, along with HIV, while others did not show a strong correlation between HIV and some heart diseases. In conclusion, after reviewing the literature, the results are inconclusive. More extensive trials focusing on the impact HIV has on heart disease are required to establish a strong correlation between HIV and heart disease to prevent morbidity and mortality.

## Introduction and background

It has been estimated that the human immunodeficiency virus (HIV) infects 1.2 million people in the United States [1]. The homosexual population, as well as minorities of all races and ethnicities, is also at risk. HIV infection cases in the United States increased to 34,800 at the beginning of 2019 [1]. After stabilizing at 37,800 in 2015, HIV infection rates declined by 8% in the majority of areas of the United States later on in 2019. However, the rate of HIV in six specific areas decreased by 9% from 2015 to 2019 [1]. Another 36,801 cases were diagnosed with HIV in the United States in 2019. States and regions did not have an equal distribution of HIV diagnoses, but the South region is mainly affected among the areas with the highest rate of newly diagnosed cases [1]. Among immune cells, a cluster of differentiation 4 (CD4) cells belongs to the T lymphocyte (T cell) subset. CD4 cells in the blood are attacked by HIV when a person has HIV. The quality and number of CD4 cells are diminished, which makes the infection rate resistance stronger. However, under antiretroviral therapy, the viral load gets suppressed and we can maintain a high number of those CD4 cells [1]. The mechanical processes underlying viral control and long-term nonprogression may differ. As a result of ongoing research efforts, novel treatment or prevention strategies for HIV infection may be developed, thus enabling the identification of host factors accordingly. However, the evidence for starting antiretroviral therapy at the point of primary infection is increasing, suggesting that it may help prevent immunological deterioration after HIV infection. At this very early stage, however, a large body of research does not examine the long-term benefits of antiretroviral therapy.

A type 1 HIV infection (HIV-1) and a type 2 HIV infection (HIV-2) are the two most common types of HIV. Acquired immunodeficiency syndrome (AIDS), known as HIV-1, causes the disease worldwide. Nevertheless, HIV-2 is only present in Africa's western and central parts. Retroviral is the prominent family of viruses that carry HIV. Because of persistent viral replication and involvement of the central nervous system, infections are typically long-term, with more extended periods of clinical latency [2]. HIV has three crucial genes, which are essential for the replication cycle: group antigen (gag), deoxyribonucleic acid polymerize (pol), and envelop protein (env) [2]. Other regulatory and accessory genes are also present in HIV-1 and HIV-2 genomes. All retroviruses, however, share the same basic structure, and a core mix of regulatory and additional genes is present in both HIV-1 and HIV-2 genomes composed of three structural genes, GAG, POL, and ENV [2]. Also, HIV-2 infection usually lasts longer before progressing to AIDS than HIV-1, as it interferes with the central nervous system [3]. Single-stranded ribonucleic acids (RNA) are the main component of retrovirus genomes. There are no differences in HIV particle structure between HIV-1 and HIV-2. GAG genes usually encode the core structural proteins (p24, p7, p6) along with the matrix protein p17, like other retroviruses. GP120 and GP41 are envelope glycoproteins recognized by surface receptors encoded by the ENV gene. The POL gene encodes the enzymes critical for the virus's replication cycle, and three reverse transcriptase enzymes that convert viral RNA into DNA [4]. The integrase incorporates Provirus DNA into the host's chromosome, while the protease cleaves large GAG and POL proteins within their components [4]. Protein 24 (p24) is the core antigen in the capsid, consisting of both matrix protein and virus membrane. Furthermore, the capsid contains two copies of HIV RNA, a nucleoprotein, three reverse transcriptases, integrase, and protease, as well as a nucleoprotein [5]. Several accessories or regulatory genes associated with HIV viruses play a critical role in modulating the replication of the virus [6].

Replication of HIV is regulated by a trimeric complex of heterodimer proteins in the viral envelope, called gp120 and gp41, which are essential for the recognition of the virus and entry into target cells [7]. At the amino terminus of the gp41 subunit, there is also a fusogenic hydrophobic peptide that facilitates viral fusion with cellular membranes [7]. When T-cell lymphocytes identify foreign antigens on site, CD4 molecules serve as coreceptors for MHCII (major histocompatibility complex 2) molecules [8]. It is believed that gp120 binds with CD4 protein, which will cause structural changes in the virus envelope, revealing a specific domain in gp120 that can bind to chemokine receptors on the cell membrane. Among HIV coreceptors, the most common are C-X-C chemokine receptor type 4 (CXCR4) and C-C motif chemokine receptor 5 (CCR5), although other potential coreceptors have been described [9]. Many cells contain the CXCR4 receptor, including T-lymphocytes, but only monocytes/macrophages, dendritic cells, and activated T-lymphocytes express CCR5. HIV-1 tropism is heavily influenced by the different expressions of these receptors on specific targets in cells [10].

In some cases, HIV-1 burdens would show that CCR5 is mainly on macrophages and CD4+ T cells that express CCR5 [11]. Once membrane fusion occurs, the virus's core uncoils into the cytoplasm, releasing viral RNA. Integrase and reverse transcriptase enzymes convert viral RNA to pro-viral DNA. Infected cells are then infected with small virions cleaved into small pieces by protease. Humans become opportunistic carriers of HIV when it affects their organs and decreases their immunity [11].

A number of scientific studies have demonstrated a link between HIV infection and cardiovascular disease (CVD). Although previous studies have found no practical evidence of HIV or its antiretroviral drugs affecting the cardiovascular system, this remains controversial [11]. HIV can impact the heart through different mechanisms. It interacts with pre-existing heart diseases such as heart failure (HF), coronary artery disease (CAD), and cardiomyopathies such as restrictive and dilated cardiomyopathies resulting in deadly complications such as sudden cardiac death (SCD), as was found in the studies in this article. It can also complicate pre-existing arrhythmias such as atrial fibrillation; also its effect on cardiac valves, which leads to heart murmurs [12]. HIV also complicates ischemic heart diseases through its impact on the cardiac muscle in pre-existing myocardial infarction, leading to fibrosis and cancer. Antiretroviral drugs can also have an impact through their side effects that affect the heart [12].

## Review

Methods

**Figure 1:**
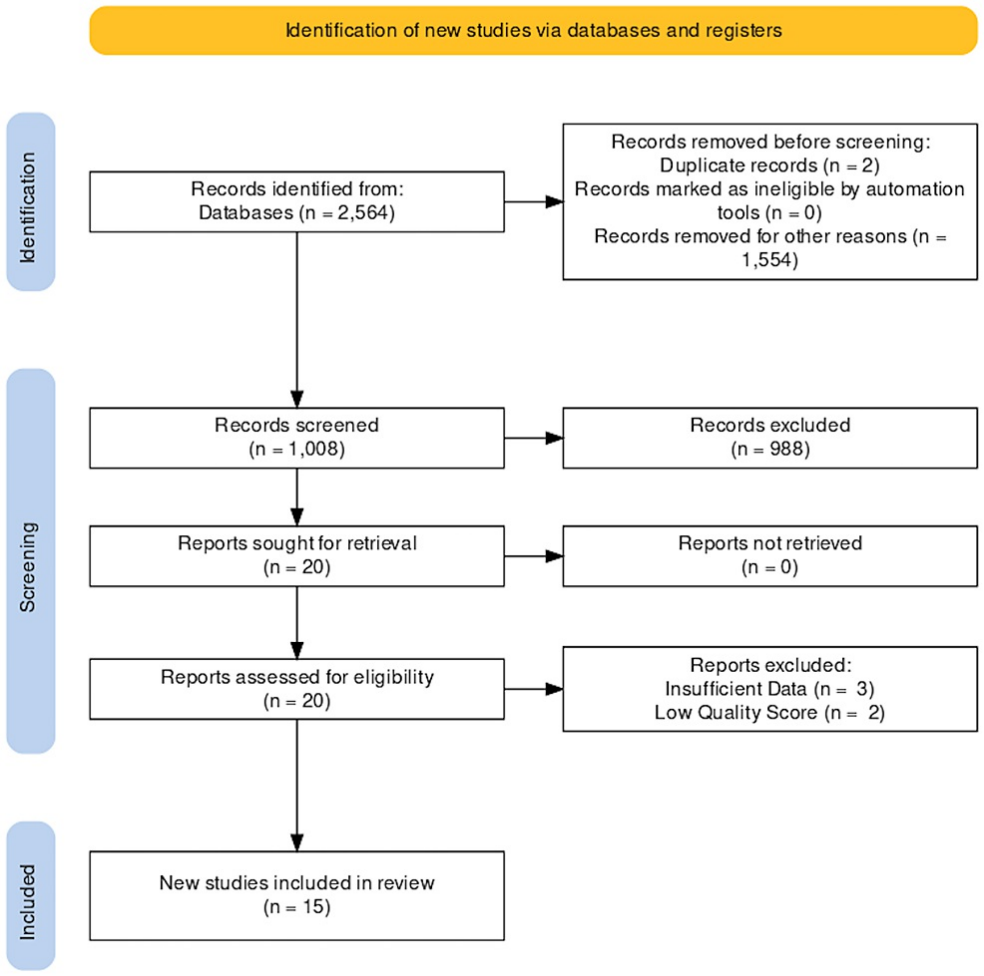
PRISMA chart for systematic and meta-analyses PRISMA, Preferred Reporting Items for Systematic Reviews and Meta-Analyses

We focused on the HIV pathophysiology and complications in pre-existing heart diseases in a systematic review by following the Transparent Reporting of Systematic Reviews and Meta-Analysis (PRISMA) 2020 guidelines [13]. Studies that met review criteria were examined and included in the study. The complete PRISMA flow diagram is shown in Figure 1.

Search Strategy

We performed a systematic search in the PubMed, Medline, PMC, EBSCO, Scopus, and POPLINE databases in order to find out the most relevant articles that we could include in our systematic review. The last date for the search was May 30, 2022. We did also search the appropriate keywords and Medical Subject Headings (MeSH) terms to create the search strategy for all available databases.

Inclusion and Exclusion Criteria

We focused on papers with a population with age group between 14 and 44 years, articles published in English language, documents from the past 10 years, papers relevant to the questions, and papers with systematic reviews such as randomized clinical trials and observational studies.

We excluded papers for populations of different ages, articles published in languages other than English, articles not relevant to the questions, papers from over 10 years ago, and papers from different species other than humans.

Analysis of Study Quality/Bias

We evaluated 20 selected studies for quality using standardized quality assessment tools, and 15 studies got qualified for the stage of medium or high quality, which was included in the review. The following assessment tools were used: (a) for observational studies, we used the Newcastle-Ottawa scale; (b) for systematic reviews and meta-analyses, we used the Assessment of Multiple Systematic Reviews (AMSTAR) tool; (c) for traditional reviews, we used the Scale of the Assessment of Narrative Review Articles (SANRA) checklist; and (d) for randomized clinical trials (RCTs), we used the Cochrane risk-of-bias assessment tool. Among all the final 20 studies, 11 were of moderate quality, four were with high-quality screens, and five were of poor quality.

Data Extraction

Data were extracted from the eligible studies according to the topic that had been chosen.

Results

A total of 2,564 articles were identified in our initial search of Medline, PubMed, PMC, EBSCO, POPLINE, and Scopus databases. A total of 1,554 articles were discarded after applying relevant filters per our eligibility criteria of the last 10 years, human studies, English papers. From 1,010 articles, two duplicates were removed. Afterward, we screened the remaining articles (n=1,008) based on titles, abstracts, full-text, and detailed inclusion-exclusion criteria. After a meticulous screening, we were left with 20 articles on our research question.

In conclusion, we got 20 studies for quality check by assessment tools. Five studies were excluded after quality appraisal, and the final 15 were included in this systematic review. We focused on systematically comparing the postinterventional adverse cardiovascular outcomes observed in patients with and without HIV infection.

Out of the 15 studies, 10 were observational, one was a randomized clinical trial, one was a comprehensive literature review, and three were systemic and meta-analyses. They included 815,062 patients with HIV of different ages ranging from 19 to 44 years. The papers studied the impact of HIV on most important heart diseases such as HF, ischemic and nonischemic heart diseases, acute coronary syndrome, myocardial fibrosis, and SCD. The studies came with moderate- to high-quality assessments. The results are outlined in Figure 1 [13].

Fifteen studies that met the quality appraisal are included in the systematic review, as discussed in Table 1 [14-28].

**Table 1:** Quality appraisal studies. ADHF, acute decompensated heart failure; AF, atrial fibrillation; AHA, American Heart Association; AIDS, acquired immunodeficiency syndrome; aOR, adjusted odd ratio; ART, antiretroviral therapy; cART, combination antiretroviral therapy; CAV, cardiac allogenic vasculopathy; CD4, cluster of differentiation 4; Cl, clearance; EF, ejection fraction; HF, heart failure; HFpEF, heart failure with preserved ejection fraction; HFrEF, heart failure with reduced ejection fraction; HIV, human immunodeficiency virus; HLH, hemophagocytic lymph histiocytosis; HT, heart transplant; MRN, medical record number; RVSP, right ventricular systolic pressure; SCD, sudden cardiac death; START, strategic timing of antiretroviral treatment; TCM, Takotsubo cardiomyopathy; UNC, University of North Carolina; VAD, ventricular assistant devices; WIHS, Women's Interagency HIV Study *EPIC is the electronic medical record system we use in hospitals

Author and year of publication	Interventions studied	Number of patients	Type of study	Result	Conclusion
Chen et al. 2021 [14]	Association of HIV with the incident of HF	8,848,569	Systemic and meta-analysis	HIV infection was positively associated with incident of HF	HF risk is twofold higher among those with HIIV than those without
Tseng et al. 2021 [15]	SCD and myocardial fibrosis, which determined by autopsy in persons with HIV	505	Observation study	People with known HIV infection were more likely than those without known HIV infection to die from occult drug overdoses (34% vs. 13%). The prevalence of interstitial myocardial fibrosis was higher in HIV-positive individuals than in those without widespread HIV infection.	Compared to those without HIV infection, HIV-positive persons were more likely to suffer SCD and myocardial fibrosis according to this study
Madan et al. 2019 [16]	Outcomes of HT in patients with HIV	41	Observation study	Among 32 patients with heart failure, we divided them into two groups; the first group included 13 people who were on ventricular assistance devices (VAD); seven of them used HeartWare type VADs, and the rest used other types. The other 19 people, however, did not have VADs.	Post-transplant survival was excellent for HIV + HT recipients. Moreover, malignancy and CAV rates for the HT population were comparable up to five years post-transplant. HT should be made available to more HIV+ transplant candidates in the future, reducing the disparity between HIV+ patients and other types of transplant candidates.
Floris-Moore et al. 2020 [17]	Computerized Adjudication of coronary heart disease events using the electronic medical record in HIV clinical research: possibilities and challenges ahead	377	Observation study	In the UNC health care system. 123 patients with coronary heart disease hospitalization were manually abstracted and events adjudicated (102 from UCHCC and 21 from WIHS), of which only 87 out of those 123 patients' records could be retrieved through EPIC* using the MRN and hospitalization dates.	In HIV longitudinal research studies and other chronic disease areas, investing in automated chart review methods is becoming increasingly important in order to facilitate more efficient outcomes assessment.
Manga et al. 2017 [18]	HIV and nonischemic heart disease	114	Observation study	Heart structure and function are affected by HIV in most cases. By causing abnormal inflammation and autoimmune responses, direct invasion of myocardial cells, increased susceptibility to opportunistic infections and tumors, as illustrated, HIV can cause hypertension in the pulmonary arteries, aortopathy, HIV-associated cardiomyopathy, and pericardial disease. Additionally, cART may have an adverse effect.	HIV-infected individuals' conceptions of heart disease have changed significantly due to cART. Regardless of CD4 cell count, HIV-infected patients should be started on cART according to START.
Abudan et al. 2020 [19]	The burden of arrhythmia in hospitalized HIV patients	2,370,751	Observation study	A 132% increase in AF is primarily responsible for the overall increase of 108% in arrhythmias over time. In older males, those with the lowest income quartile, and those admitted with no choice in their treatment, arrhythmias are more prevalent. In-hospital mortality rates for patients with arrhythmias were 9.6%. As a result of the reduction in in-hospital mortality among patients with arrhythmias over time, 43.8% of patients have survived.	Patients hospitalized with HIV are significantly more likely to suffer from arrhythmias and die from them. Patients hospitalized with HIV are most likely to suffer from AF.
Sinha et al. 2020 [20]	Association of low CD4/CD8 ratio with adverse cardiac mechanics in lymphopenic HIV-infected adults	270	Observation study	Among the overall study population included in primary analyses, we noticed a higher RVSP with a lower CD4/CD8 ratio both at baseline and at echo post-ART.	It warrants further study that a reduced CD4/CD8 ratio is associated with a higher RVSP in HIV patients with very low CD4 counts and may impact HIV-associated HF.
Wongcharoen et al. 2013 [21]	Heart rate variability and heart rate turbulence in HIV-infected patients receiving cART	123	Randomized clinical trial	Although baseline characteristics of HIV-infected patients and control subjects were similar, HIV-infected patients had significantly higher triglyceride and glucose levels at fasting.	A study conducted on HIV-infected patients receiving cART revealed autonomic dysfunction. This group of patients showed an overall decrease in HRV.
Bundhun et al. 2017 [22]	HIV impact on cardiovascular outcomes following percutaneous coronary intervention	2268	Systemic and meta-analysis	Patients with HIV or without HIV did not have a significantly higher mortality rate.	Found similar mortality post-coronary intervention among those infected with HIV or not infected.
Shah et al. 2018 [23]	Global burden effect of atherosclerotic cardiovascular disease in people with HIV	793,635	Systemic and meta-analysis	The risk of cardiovascular disease was 2.16 higher than those without HIV.	People living with HIV are twice to develop cardiovascular disease.
Boccara et al. 2020 [24]	HIV infection and long-term residual cardiovascular risk after acute coronary syndrome	298	Observation study	An average of 12 years standard deviation and 8 years [mean, standard deviation, 8 years] of known HIV infection was encountered in HIV-positive individuals, with median CD4 cell counts of 52 (quartile 1 = 52, quartile 2 = 210) and 462 (quartile 1 = 270, quartile 2 = 640) per μL, respectively.	Those living with HIV are more likely to experience recurrent acute coronary syndrome, primarily due to new lesions in the coronary arteries, especially during the first year, and are more likely to suffer from HF that requires hospitalization.
Yen et al. 2019 [25]	Association of HIV infection and ART with SCD	121,530	Observation study	There were 5,342 deaths out of 121,530 patients (24,306 HIV+ and 97,224 matched controls) in a mean follow-up period of 5.86 years; among them, 150 (0.12%) died of SCD. In 150 SCD cases, 97 (64.7%) were attributed to the HIV-positive population and 53 (33.3%) to the control population, which translates into an incidence of 68.31 per 100.000 person-years for HIV positives and 9.31 per 100.000 person-years for controls (p = 0.001).	As a risk factor for SCD, HIV infection appears to be independent. People living with HIV receiving ART have low rates of these diseases.
Estroza et al. 2022 [26]	Patterns of HF in people with HIV	305	Observation study	It is estimated that 17% of HF patients have HF, 33.5% have HFrEF, 23.2% have HF with borderline EF, and 40.4% have HFpEF.	According to American Heart Association and American College of Cardiology guidelines, the early assessment of the long-term risk factors in young adults like any chronic diseases (diabetes mellitus, high cholesterol level, hypertension) which lead to Ischemic heart disease could help and guide them to modify their lifestyle by doing exercises and regular checks and follow-ups
Almani et al. 2021 [27]	Impact of HIV infection on the outcome of hospitalized patients with ADHF analysis of National Inpatient Sample	179,325	Observation study	Compared to their counterparts, HIV patients had similar odds of developing cardiovascular shock (aOR: 0.84, 95% CI: 0.424-1.662, p=0.615) whereas infectious endocarditis caused higher odds of death (aOR: 4.9, 95% CI: 1.627-14.561, p=0.005).	Compared to patients without HIV infection, patients with HIV infection admitted for ADHF had similar inpatient mortality. However, patients with HIV infection were more likely to suffer from infectious complications.
Ullah et al. 2019 [28]	TCM secondary to HLH in HIV patients	9	Comprehensive literature review	The first case of HLH presented as TCM was found in a comprehensive literature review.	When encountering such patients, it is important to keep in mind that HIV may precipitate TCM.

Discussion

**Figure 2:**
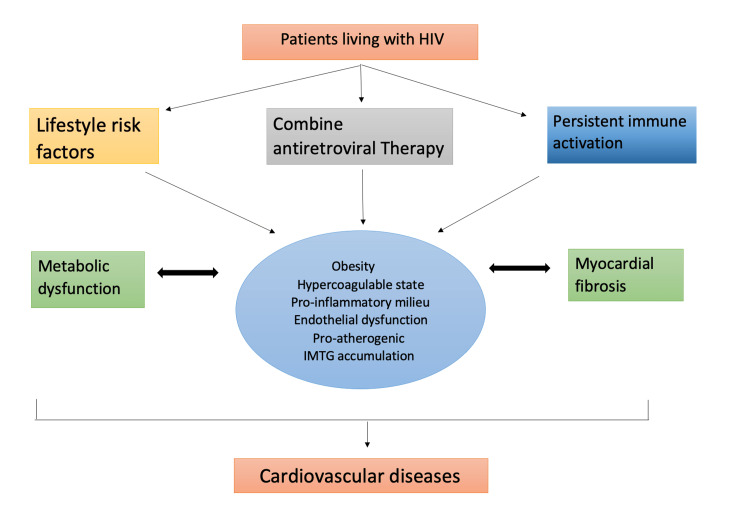
HIV and antiretroviral impact on the heart IMTG, intramuscular triglycerides

Our systematic review of 15 studies shows that the impact of HIV may differ on the heart and can trigger many heart diseases, as shown in Figure 2. We have chosen two different pathways, as shown in the figure.

Pathophysiological mechanisms showing HIV’s effect on different heart diseases

The study by Chen et al. involving 8,848,569 patients showed that HIV infection is implicated as an independent risk factor for developing atherosclerotic cardiovascular disease. Viral infection contributes to atherosclerosis by stimulating the proliferation of smooth muscle cells in the vessels, activating the immune system, causing chronic inflammation, altering coagulation, and causing lipid imbalances [14]. Our findings suggest that these factors may increase the likelihood of developing coronary heart disease and HF. As a result, interleukin (IL)-1b, tumor necrosis factor-alpha (TNF-a), and free radicals are upregulated in cardiac endothelial cells simultaneously as HIV can directly invade and remain latent there [14]. HF may also be accompanied by pericardial adipose tissue increase due to chronic inflammation and immune activation caused by these cytokines. Clinical and public health approaches must be designed to better target HF risk stratification, screening, and early preventative treatments for HIV-positive persons to raise awareness of HF risk. Patients with HIV who exhibit no cardiovascular risk factors should be treated with cardiovascular health care as part of their clinical care [14]. There are not enough data to suggest that prevention and treatment approaches for HF should differ fundamentally from those for the general population. According to the American Heart Association and American College of Cardiology guidelines, longer-term risk assessment among younger adults will guide lifestyle modification intensity and type decisions. Consequently, preventing or delaying HF by identifying and managing traditional risk factors will play a crucial role [14]. In spite of this, novel treatments aimed at decreasing the long-term proinflammatory response associated with HIV infection might be particularly effective in preventing atherosclerotic CVD and HF. Still, caution must be exercised to prevent opportunistic infections from developing [14].

In contrast, the study by Tseng et al. included 505 patients. The author found that HIV-positive individuals are at greater risk of developing interstitial cardiac fibrosis than HIV-negative individuals [15]. As assessed by late gadolinium enhancement and decreased cardiac function, magnetic resonance imaging of HIV-positive individuals without any symptoms shows an increased incidence of myocardial fibrosis. Survivors of cardiac arrest and people with nonischemic dilated cardiomyopathy also show higher late gadolinium enhancement. In addition, besides ejection fraction, it is a good predictor of all-cause mortality. They found histologic confirmation among HIV-positive patients with myocardial fibrosis that this condition may lead to sudden death from arrhythmia [15]. The most common causes of cardiac fibrosis are myocardial infarction, hypertension, HF, and valvular diseases. We found that the proportion of individuals who had these conditions was so much similar in severity whether they had HIV or not, suggesting that HIV-related factors contribute to myocardial fibrosis in addition to myocardial infarction. The prevalence of interstitial fibrosis was also higher in persons with HIV infection than in those with CAD, which is usually attributed to CAD [15]. On the other hand, Madan et al. conducted a study in 2019 including 41 patients. Transplantation of hearts performed on HIV-positive recipients is on the rise, which is encouraging. However, most of the centers in the United States (>80%) do not perform this procedure [16]. HT provides the same benefits to HIV-positive patients as to non-HIV-positive patients, according to previous reports and our current analysis. Our goal is to help increase the number of centers that offer HT to HIV-positive patients based on the scientific evidence we provide here. It might be time now, according to some, when HIV-positive organs would be considered for patients who are HIV-positive [16].

In contrast, the study by Floris-Moore et al. included 377 patients. Although hospital electronic medical records (EMRs) already possess research capabilities, extensive collaboration with institutional information technology and bioinformatics professionals will be necessary to maximize their capability of detecting clinical outcomes and adjudicating them using computerized tools. [17]. To ensure the validity of event adjudication in clinical research, it is essential that the data are complete. EMRs will need uniform coding and mapping for health care systems with more than one clinical site. Since billing practices, which influence the final diagnosis codes associated with hospitalization, vary considerably between hospital systems, this challenge is heightened in multicenter studies, such as the WIHS (Women's Interagency HIV Study). Improved research study design and data retrieval and linking processes will be needed in order to overcome these limitations [17]. For investigators to understand the limitations of electronic data and to ensure that data retrieval provides both accurate and comprehensive data concerning the research question, early collaboration with data analysts with EMR-specific expertise will be crucial during the development of the study [17]. Developing a standard process for retrieving data from all encounters that occur during a single hospitalization event is essential for addressing diagnoses such as myocardial infarction, which require the assessment of test results over time. As an example, the troponin levels and electrocardiogram (ECG) results from an ED may be linked to admission to the hospital, which may exist separately in the EMR [17].

Manga et al. conducted a study in 2017 involving 114 HIV-positive patients, which showed different aspects of HIV involvement, including pericardial, aortopathy, cardiomyopathy, and pulmonary artery hypertension [18]. It is clinically reasonable to use standard HF therapies, such as automatic implantable cardioverter-defibrillators and cardiac resynchronization therapy, in patients with HIV-associated cardiomyopathy despite the lack of randomized controlled trials. It is still recommended to initiate combination antiretroviral therapy in all patients who are not on antiretroviral therapy, as restoration of immune status and suppression of the virus may help alleviate the symptoms of HIV-associated cardiomyopathy [18]. Compared to HIV-negative patients, HIV-positive patients undergoing cardiac surgery are probably at no greater risk. The current guidelines should be followed in order to offer device therapy to these patients. Heart transplantation can be considered individually in HIV-positive patients with refractory HF [18].

An observation study by Abudan et al. involved 2,370,751 incidences of arrhythmias in HIV patients versus non-HIV patients [19]. The author found that among HIV-related hospitalizations, the following variables were independently related to arrhythmia: male gender, older age, higher income, later admission year, and comorbidities including congestive HF, obesity, CAD, renal failure, hypertension, chronic pulmonary disease, a history of myocardial infarction, and peripheral vascular disease [19]. On the other hand, the study by Sinha et al. involved 270 patients. The authors found that post-ART echocardiography showed higher right ventricular systolic pressure in subjects with lower CD4/CD8 ratios after antiretroviral therapy initiation [20]. It is associated with a higher risk of cancer when CD4 counts are low in HF in the broader HIV population, according to recent studies. The mechanistic and clinical studies of HIV-related CD4 lymphopenia need to determine whether it is a cause of cardiac dysfunction or HF or a proxy marker for other virological and inflammatory factors leading to HF [20].

**Figure 3:**
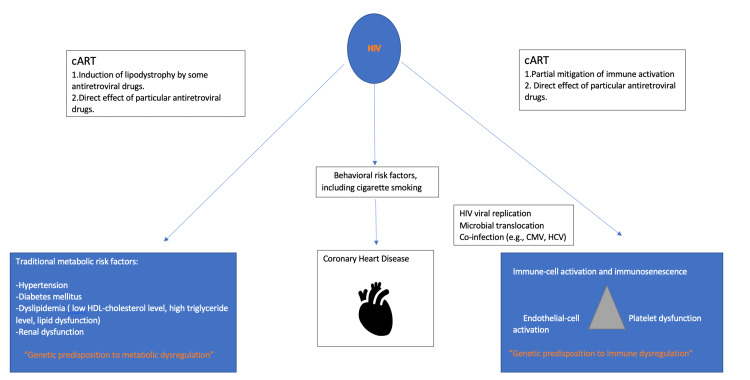
The impact of HIV and cART on the heart (coronary heart disease). cART, combine antiretroviral treatment; CMV, cytomegalovirus; HCV, herpes simplex virus

As for this study, a randomized clinical trial conducted by Wongcharoen et al. included 123 patients. The author found that several possible factors have been proposed to explain the pathophysiology of autonomic dysfunction among HIV-infected individuals [21]. HIV itself has a neurotropic effect, which is the first explanation. We identified only a weakly positive correlation between autonomic function and immune status, suggesting that this factor plays only a limited role in determining cardiac autonomic dysfunction [21]. Secondly, longstanding antiretroviral therapy treatment may cause autonomic dysfunction, as shown in Figure 3. As early diabetes and metabolic syndrome demonstrate, combine antiretroviral treatment (cART) could also damage parasympathetic function. We were unable to locate the object despite our efforts an association between different types of cART and autonomic function.

Complications caused by HIV in pre-existing cardiac diseases and cardiac complications of HIV in healthy individuals 

A systematic review and meta-analysis by Bundhun et al. involved 2,268 patients [22]. One to three years after percutaneous coronary intervention (PCI), no differences were found in cardiovascular outcomes between HIV-infected and HIV-uninfected patients [22]. In contrast, a systematic review with a meta-analysis conducted by Shah et al. included 793,635 patients. The author found out that the increased risk of CVD in people living with HIV is due to accelerated atherosclerosis due to chronic infection and the increased prevalence of traditional risk factors [23]. As for this study, an observational study conducted by Boccara et al. involved 298 patients. The authors found that there are strong correlations between chronic HIV infection and chronic inflammation, including a first recurrent event, mostly myocardial infarction, and they result in new coronary lesions in two-thirds of patients [24]. Similarly, HIV-positive patients' cardiovascular residual risk profile was worse after acute coronary syndrome despite having a lower rate of smoking cessation and a smaller decrease in total cholesterol [24].

On the other hand, an observational study conducted by Yen et al. involved 121,530 patients. The authors found that the prevalence of SCD was lower in HIV-positive people receiving antiretroviral therapy than in those not receiving the treatment [25]. HIV people may be at a lower risk of SCD if they receive antiretroviral therapy that inhibits virus replication and prevents endothelial dysfunction and atherosclerosis. As a result, HIV patients are less likely to develop cardiomyopathy, reducing their risk of developing SCD [25].

**Figure 4:**
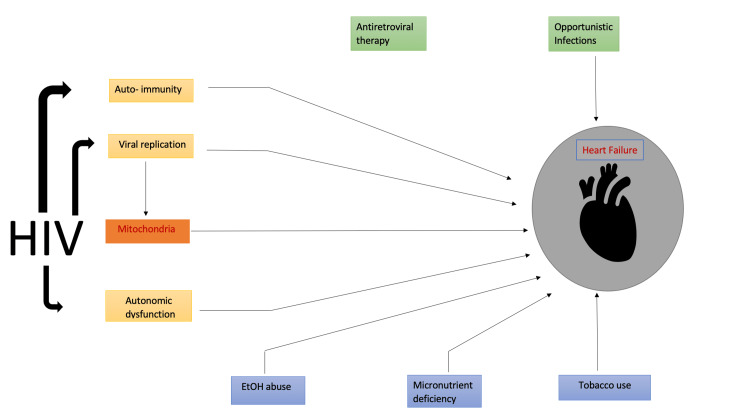
How HIV affects and increases the risk of heart failure. EtOH, ethyl alcohol

Estroza et al. conducted an observational study in 2022 involving 305 patients. According to AHA 2020, HIV-positive patients with heart failure with reduced ejection fraction (HFrEF), patients with heart failure with preserved ejection fraction (HFpEF), and patients with borderline ejection fraction (EF) had a 2.2% higher prevalence of HF than uninfected patients. However, there were no significant differences between HIV-positive patients with HFrEF, HFpEF, and HF with borderline EF in terms of traditional and HIV-related risk factors [26]. Identifying the mechanisms related to the development of HFrEF versus HFpEF, as well as risk stratification for early risk-factor intervention, is crucial to decreasing the risk of HF in high-risk populations, as shown in Figure 4 [26].

In contrast, an observational study by Almani et al. found that 975 of the 179,325 patients with acute decompensated HF (ADHF) were HIV-positive [27]. There was a 0.84 (95% CI 0.018 - 1.029, p=0.053) adjusted odds ratio for inpatient mortality in those with ADHF with HIV compared to those without HIV [27]. The inpatient mortality rates for HIV-infected patients admitted for ADHF were similar to those for HIV-negative patients, and they are more likely to develop infectious complications.

The final study was conducted by Ullah et al., which included only nine patients. It was a comprehensive literature review. According to the study, only nine cases of cardiac complications were reported to be associated with hemophagocytic lymphocytosis (HLH) [28]. Four of the nine patients reported were adults, while five were children. In all but two cases, there was fever, and in two cases, there was dyspnea. A biopsy was available for confirmation in only one case, but four cases were diagnosed with myocarditis. We were not the only ones to diagnose Takotsubo cardiomyopathy because one other patient presented with fever and fatigue as opposed to ours [28]. The presence of hypertrophic obstructive cardiomyopathy in two instances associated with HLH is interesting to note. It was eventually possible for most patients to recover. There has not been any case reported in which HIV was the inciting event for HLH nor have any cases been reported in which cardiogenic shock with global hypokinesia was the presenting symptom [28].

Limitations

In most of the studies we included, we had few sample sizes. One study was having only nine patients, being the lowest sample size. We did not specify genders and did not go with ages different from our inclusion criteria. We also did not specify the locations were those studies conducted, whether being all in the United States or different countries. Meanwhile, in some studies, we still could not determine how HIV has the burden of complicating heart diseases because we did not have that much data. We could not find more than one randomized clinical trial for our article, because of the topic being not that much common.

## Conclusions

We came up with two different pathways in which HIV can impact the heart through pathophysiological pathways and complications triggers. We explained all of them in 15 studies. One study was a randomized clinical trial, and all others were observational studies. As for HIV pathophysiology, we found how HIV interacts with the immune system and leads to decrease CD4 cells count, how it interferes with the central nervous system to become latent for life, and how in patients with HF or myocardial infarction it will lead to fibrosis or cancer of the cardiac muscles, which leads to SCD. However, we found that it has no effect of heart procedures such as PCI or heart transplant as we compared patients with and without HIV who had undergone procedures. However, we found how HIV has an effect on accelerating pre-existing cardiac conditions such as atherosclerosis, HFrEF, or HFrEF, and also how ARTs have an effect on the metabolic and the parasympathetic functions. Although this topic is uncommon, the effect of HIV on the heart should have more studies and research in the future to go into deeper explaining the effect of HIV as a virus or the side effect of the antiretroviral treatments. This topic deserves more spotlight, and thus we can increase the burden of preventive strategies and improve people’s awareness of how vital and severe HIV infection is.
